# Role of Interleukin-1 family in bone metastasis of prostate cancer

**DOI:** 10.3389/fonc.2022.951167

**Published:** 2022-09-27

**Authors:** Yuanhao Tong, Yinghao Cao, Tianzhe Jin, Zhengwei Huang, Qinyuan He, Min Mao

**Affiliations:** ^1^ School of Medicine, Zhejiang University, Hangzhou, China; ^2^ Department of Orthopedics, Shanghai General Hospital, Shanghai Jiao Tong University School of Medicine, Shanghai, China; ^3^ Department of Gynecologic Oncology, Women’s Hospital, School of Medicine, Zhejiang University, Hangzhou, China; ^4^ Organization Department, Suzhou Traditional Chinese Medicine Hospital, Suzhou, China

**Keywords:** interleukin-1 family, inflammation, cancer immunity, prostate cancer, bone metastasis

## Abstract

Prostate cancer (PCa) is one of the most fatal diseases in male patients with high bone metastatic potential. Bone metastasis severely shortens overall survival and brings skeletal-related events (SREs) which reduces the life quality of patients, and this situation is currently regarded as irreversible and incurable. The progression and metastasis of PCa are found to be closely associated with inflammatory cytokines and chemokines. As pivotal members of inflammatory cytokines, Interleukin-1 (IL-1) family plays a crucial role in this process. Elevated expression of IL-1 family was detected in PCa patients with bone metastasis, and accumulating evidences proved that IL-1 family could exert vital effects on the progression and bone metastasis of many cancers, while some members have dual effects. In this review, we discuss the role of IL-1 family in the bone metastasis of PCa. Furthermore, we demonstrate that many members of IL-1 family could act as pivotal biomarkers to predict the clinical stage and prognosis of PCa patients. More importantly, we have elucidated the role of IL-1 family in the bone metastasis of PCa, which could provide potential targets for the treatment of PCa bone metastasis and probable directions for future research.

## Introduction

In 2020, PCa is the fifth leading cause of cancer death among men, with an estimated 1.4 million new cases and 375,000 deaths worldwide. The age-standardized incidence and mortality rate of PCa is 30.7% and 7.7% respectively (1). Generally, patients with early-stage and low-risk PCa can be treated with prostatectomy or radiation, while patients with advanced stages of PCa could be treated with androgen deprivation therapy (ADT), as androgen and androgen receptor(AR) were found to play an important role in the progression of PCa (2). However, ADT is only effective in the early course of tumors. The majority of advanced cancer patients, which are called castration-resistant prostate cancer (CRPC) or neuroendocrine prostate cancer (NEPC), still have no response to currently available ADT (3) (4). As reported, the mean survival time of the patients with the metastasis of CRPC is approximately 14 months (range 9-30) (5).

As reported, 65-75% of advanced PCa patients have bone metastasis (6), furthermore, the rate of metastasis to bone even reaches 90% in CRPC patients (7). The 5-year survival rate of PCa without metastasis is nearly 100%, however, once metastasis, as reported, the 5-year survival rate is merely 28.2%, and the mean survival time of patients with bone metastasis is only 19 months in PCa (8). Bone metastasis often cause SREs, including debilitating bone pain, nerve root or spine cord compression, vertebral fractures, hypercalcemia, and bone marrow infiltration that leads to cytopenia (9), resulting in a severe decline of patients’ life quality. PCa bone metastasis are predominantly present in the axial bones including the spine(90%), sacrum and pelvis (10). The spine is the major metastatic site of bone in PCa.

Currently, bone metastasis targeting agents include bisphosphonates and denosumab. They are usually applied to reduce SREs, such as pain and fracture, through modeling the bone microenvironment. However, the current palliative treatments could not be applied to prevent bone metastasis and improve the prognosis of metastasis PCa. Immune therapy, which is considered a potential treatment option for metastatic PCa, recently has received increasing attention. For example, Sipuleucel-T, a cellular immunotherapy which has already been approved by the US Food and Drug Administration (FDA), could improve the overall survival of PCa, while it has no effect to suppress the progression of PCa (11). In addition, as reported, immune checkpoint inhibitors, including anti-PD-1 and anti-CTLA4, also do not function well in metastatic PCa (12). Although single CTLA4 blockade and anti-PD-1 therapy are not effective, they show a curative effect when combined with radiotherapy or chemotherapy (13). This could be explained by the “Cancer Immunoediting” theory that tumor cells that can avoid immune recognition will survive and escape from immune therapy (14). Additionally, validated predictive biomarkers are urgently needed to assess patients’ responses to treatment.

Considering the poor prognosis of bone metastatic PCa, further research should be performed to elucidate the potential mechanism of the immune environment in PCa to detect more curative and effective immunotherapy.

## Mechanism of PCa metastasis

### Inflammation and PCa bone metastasis

Several types of research have evidenced that inflammation is significantly correlated with cancer (15–17). It is widely acknowledged that inflammation exerts a vital role in maintaining homeostasis as a defense mechanism against infection and injury. However, when the homeostasis is broken, exaggerated inflammation can also promote tumorigenesis, and progression and metastasis (18). In most occasions, systemic inflammation characterizes the early stages of the metastatic cascade.

Accumulated evidence has proven that inflammation promotes bone metastasis through various mechanisms. Pro-inflammatory cytokines and other immune agents including chemokines and selectins, as the key components of tumor microenvironment, exert a vital role in the survival, proliferation and metastasis of tumor (19, 20). The microenvironment determines the metastatic place by supporting the formation of a pre-metastatic niche and bone colonization, metastatic dormancy and reactivation (21). The process of metastasis begins with cancer cells invading adjacent tissue and the formation of EMT. Previous research found that MMPS induced by Myeloid-derived suppressor cells (MDSCs) can remodel the extracellular matrix and promote tumor migration (22), and MDSCs can also mediate suppression of anti-tumor response (23). It was evidenced that cancer stem cells(CSCs) activated by inflammatory signaling can promote bone metastasis and induce resistance to treatment (24). Tumor-associated macrophages (TAM) are also reported to be closely involved in PCa bone metastasis by promoting angiogenesis and immune evasion (18). Furthermore, bone marrow adipocytes, which are closely associated with inflammation, are found to favor bone metastasis by attracting invading tumor cells and by activating the NF-κB pathway and producing inflammatory cytokines and chemokines (18, 25). Another significant component of the inflammatory environment, cancer-associated factors (CAFs), also exert a crucial role in bone metastasis directly or indirectly. For example, stromal-derived factor 1 (SDF-1/CXCL12) secreted by CAFs promotes bone metastasis by interacting with cancer cells through CXCR4, while CAFs-secreted epidermal growth factor receptor (EGFR) can promote bone metastasis by directly interacting with tumor cells (26).

Inflammatory mediators also play vital roles in the progression and metastasis of PCa, with some of them promoting tumor growth while others exerting antitumor function. For example, TNF-α, IL-6, IL-8, and IL-23 mediate inflammation resulting in tumor growth, and TGF-β and CXCL12/CXCR4 mainly promote metastasis. Inversely, IL-10 and IL-12 suppress tumor growth by inducing IFN-γ and activating T and NK cells (27).

**Figure 1:**
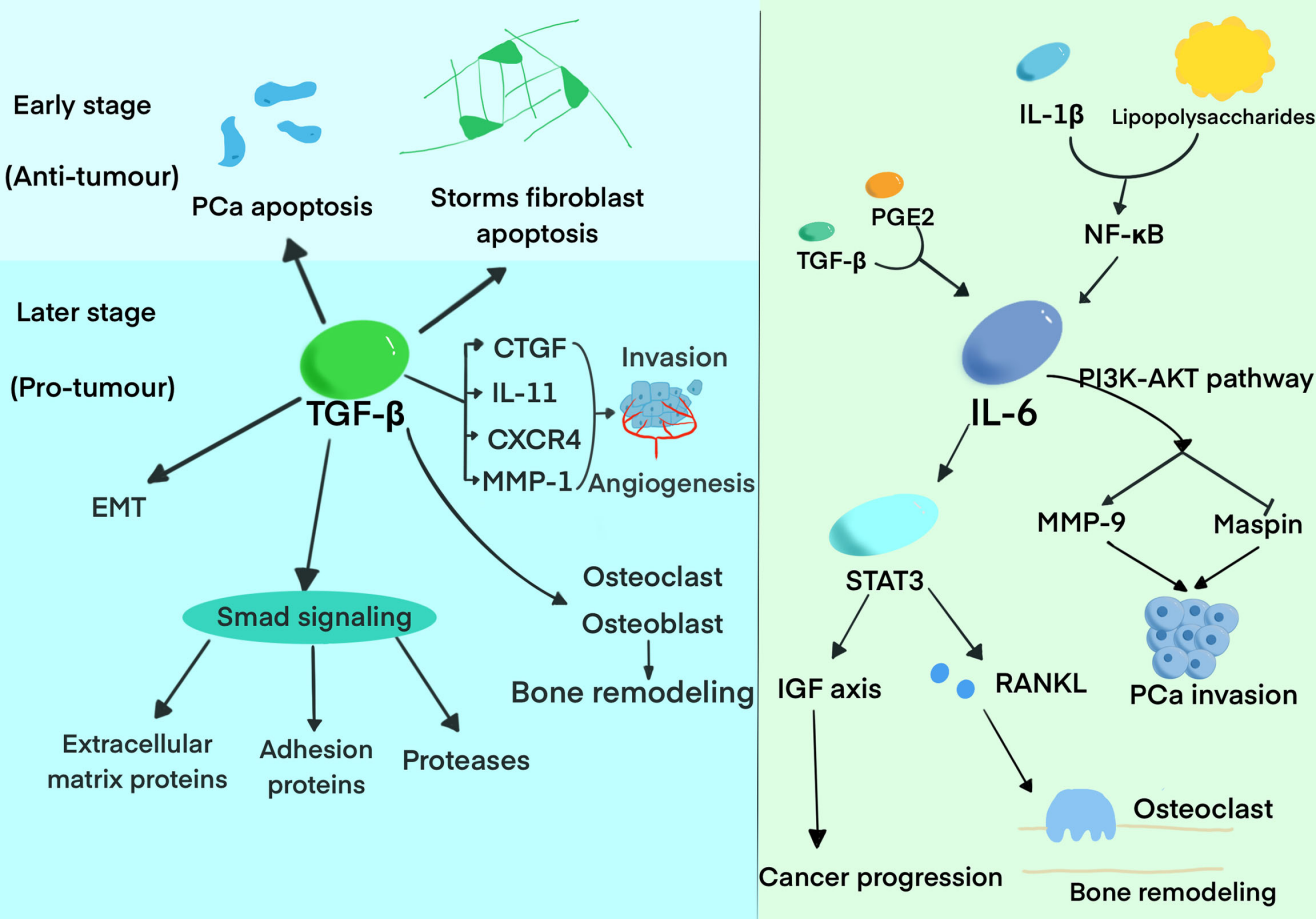
The roles of TGF-β and IL-6 in the progression and bone metastasis of PCa. The diagram summarized the pivotal functions of TGF-β and IL-6 has in the progression of bone metastasis.

IL-6, a significant proinflammatory cytokine, was proved by accumulating evidences that it exerts a crucial role in tumor cell proliferation, colonization, angiogenesis, and bone metastasis (**Figure 1**). The expression of IL-6 can be upregulated by inflammation in the bone, and two main pathways play vital roles in the process. Prostaglandin E2(PGE2) and TGF-β directly upregulate IL-6, while IL-1β and lipopolysaccharides stimulate IL-6 production *via* NF-κB activation (28). It was demonstrated that IL-6 has an important role on PCa cell growth, metastasis, and bone remodeling mainly through JAK-STAT3, MAPK and PI3K–AKT pathways (29). IL-6 can promote the progression and metastasis of PCa through stimulating STAT3 expression and upregulating the paracrine insulin-like growth factor(IGF) axis (30). It was also reported that IL-6 can induce the expression of RANKL after activating STAT3, causing a direct stimulation of osteoclast activity, and finally resulting in bone destruction (31). IL-6 and oncostatin-M(OSM) are found to promote PCa cell invasion through the PI3K/AKT pathway (32). In addition, a recent study demonstrated that IL-6 and IL-6R expression was positively correlated with the level of plasma PSA in PCa. Therefore, these studies collectively elucidated that IL-6 could act as a potential inflammatory biomarker in the progression and metastasis of PCa (33).

Increasing evidence demonstrated that TGF-β probably exerts a dual function, promoting or suppressing the progression and metastasis of PCa, depending on the tumor stage. TGF-β suppresses tumor growth in the early stage of PCa development by inhibiting cell proliferation and inducing apoptosis. TGF-β not only exerts its growth inhibitory effect on target cells but also functions on stromal fibroblasts and inflammatory cells. Meanwhile, TGF-β was regarded as the key suppressor of tumor infiltrating macrophages, NK cells, and effector T cells thus promoting immune tolerance (34). While it exerts an inverse effect in the later stages, including epithelial-to-mesenchymal transition (EMT), immunosuppression, extracellular matrix degradation and angiogenesis (35). Accumulating evidence proved that high expression of TGF-β is associated with tumor metastasis and worse prognosis in PCa patients (36). TGF-β was found to induce extracellular matrix proteins, cell adhesion proteins, and proteases in PCa mainly through Smad signaling (37). It was demonstrated that TGF-β exerts the function of bone modeling by regulating the differentiation, proliferation, and function of osteoblast and osteoclast (38). Research has also reported that TGF-β can regulate the expression of connective tissue growth factor (CTGF), IL-11, CXCR4, and MMP-1 to promote bone metastasis by inducing angiogenesis, invasion, and homing to bone (39, 40). Furthermore, TGF-β can promote bone metastasis by regulating the expression of genes involved in bone metastasis (41). Interestingly, prostate transmembrane protein androgen-induced 1 (PMEPA1), one of the genes induced by TGF-β, inhibits TGF-β signaling and bone metastasis in negative feedback (42).

### Immune evasion in prostate cancer

Immune evasion plays a crucial role in PCa progression and metastasis, which involves many factors. For example, prostate cancer cells express few tumor antigens, which results in a low immune response. Absence of human leukocyte antigen (HLA) class I also leads to impaired cytotoxic T lymphocyte and tumor survival. Additionally, the expression of immune checkpoint ligands on cancer cells, such as PD-L1, which binds to PD-1, causes metastasis and a low response to immune checkpoint inhibitors (43).

Several factors function in the process of immune evasion. For example, EMT was found to be associated with upregulated indoleamine 2,3-dioxygenase-1(IDO1) and an increased number of regulatory T cells, which promotes immune evasion (44). IFN-γ can induce the expression of PD-L1 by activating NF-κB and RelB nuclear translocation. And PD-L1 can inhibit NK cells and cytotoxic T lymphocytes, suppressing the effect of immune therapy (45). Androgen receptor plays a vital role in immune evasion by regulating PVR, an immunological checkpoint gene(also named CD55), through its enhancer (46). Dickkop-1(DKK1) plays a vital role in immune evasion in double-negative prostate cancer(DNPC), While ET1 induced by IL-1 can suppress DKK1 (47, 48).

MDSCs also play a crucial role in immune evasion. MDSC is mainly regulated by factors secreted by tumor cells, such as stem cell factor(SCF) and vascular endothelial growth factor(VEGF) which increase the number of MDSCs and inflammatory cytokines and chemokines such as IL-4, IL-6, IFN-γ, and IL-1β which suppress MDSC. IDO can mediate the recruitment of MDSCs relying on Treg (49). The mechanisms of MDSCs inducing immune evasion include decreased expression of l-selectin by T cells, upregulation of oxidative stress, induction of immunosuppressive cells such as regulatory T(Treg) and T helper(Th)17 cells which inhibit the normal tumor-suppressing function of T cells (50). MDSCs can also regulate tumor angiogenesis and remodel the microenvironment through VEGF, bFGF, Bv8, and MMP-9 to promote tumor progression (51). Research has shown that bisphosphonates can inhibit the mobilization of MDSCs (50).

Moreover, IL-33, a member of the IL-1 family, was reported to be involved in immune surveillance, and its absence can lead to immune evasion through “Cancer Immunoediting” (52).

As the IL-1 family are involved widely in inflammation and are associated with a large number of proinflammatory cytokines, they are found to be closely associated with PCa progression, and many further kinds of research on the role of IL-1 in PCa and bone metastasis have been carried out.

## IL-1 family and PCa progression

**Figure 2:**
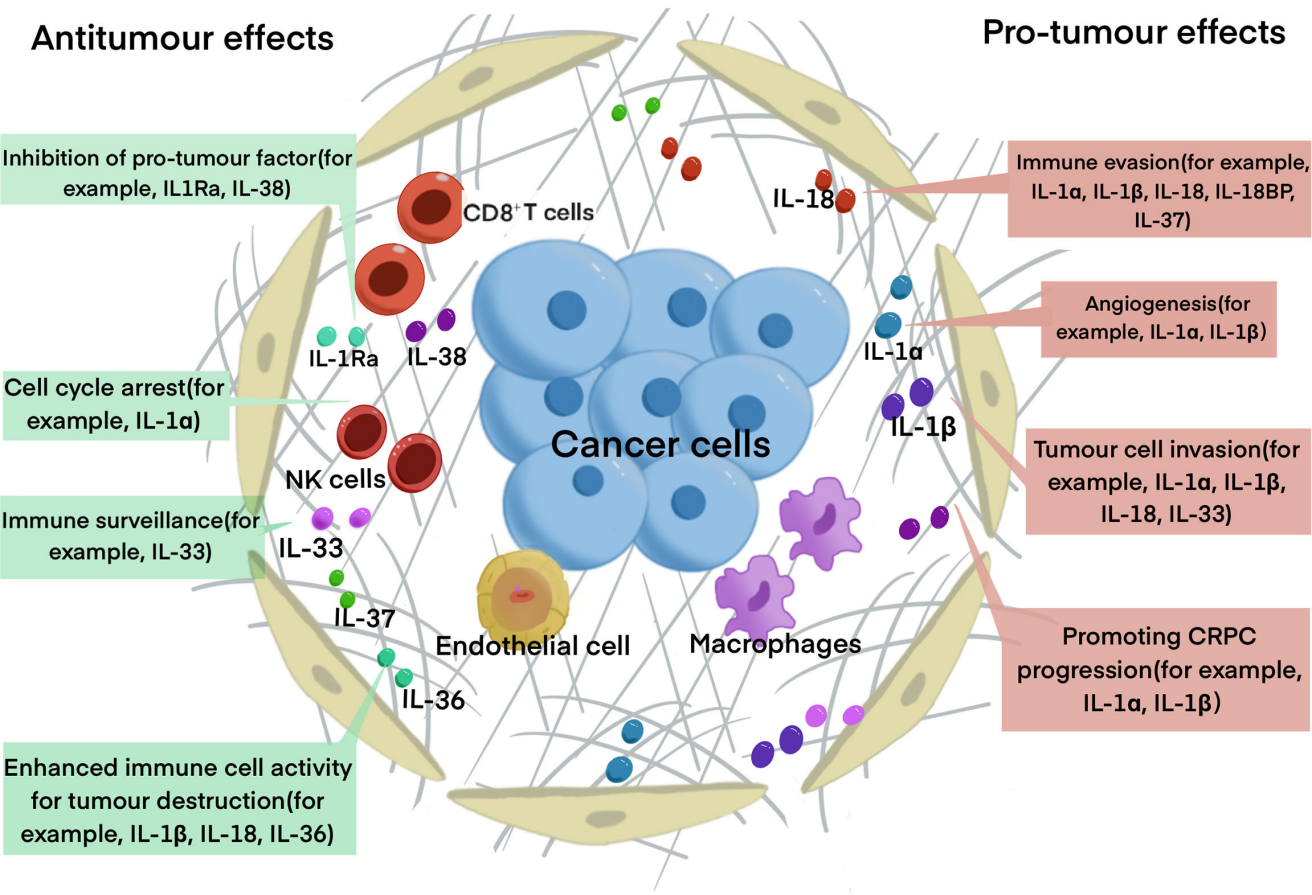
The roles of IL-1 family in PCa progression. The diagram summarized the major function of IL-1 family has in PCa progression. Some of the cytokines, for example, IL-1Ra, IL-33 and IL-37 have anti-tumor function, IL-1α and IL-1β have pro-tumor function, while IL-18 and IL-33 have dual functions.

The IL-1 family currently comprises of nine proinflammatory cytokines (IL-1α, IL-1β, IL-18, IL-33, IL-36α, IL-36β, IL-36γ) as well as two anti-inflammatory cytokines (IL-1RA, IL-36RA, IL-37, IL-38). Based on IL-1 consensus sequence and the signaling receptor chain, IL-1 family can be divided into 3 subgroups: secreted molecules with agonistic activity (IL-1α, IL-1β, IL-18, IL-33, IL-36Ra, IL-36α, IL-36β, IL-36γ), receptor antagonists (IL-1RA, IL-36Ra, IL-38), and an anti-inflammatory cytokine (IL-37). They can also be categorized into 3 subfamilies according to the length of the precursor and the length of the pro piece for this precursor: IL1 subfamily (IL-1α, IL-1β, IL-33), IL-18 subfamily (IL-18 and IL-37), and IL-36 subfamily (IL-36RA, IL-36α, IL-36β, IL-36γ, IL-38), while IL-1RA is not concluded in the subfamilies (53, 54). IL-1 family was found to promote cancer progression through interacting with inflammatory cytokines and downstream pathways, while some members of it exert anti-tumorigenic functions (55) (**Figure 2**).

There are two major agonistic IL-1 ligands, IL-1α and IL-1β, and one antagonistic ligand IL-1RA, and all of them can be activated by NF-κB. Both IL-1α and IL-1β were shown to exert a dual functions in promoting and suppressing tumor progression. It was noticed that overexpression of IL-1α may have anti-tumor effects (56). IL-1α could also suppress the progression of the tumor by inducing G0-G1 phase cell cycle arrest in PCa (56). IL-1β was reported to induce Th1 and Th17 to strengthen the anti-tumor effect. IL-1β also exerts anti-tumor effects, which can prevent metastatic cells from colonization in the metastatic place, thus inhibiting metastasis (55). Studies also found that IL-1α and IL-1β play a role in cancer eradication mediated by tumor-specific Th1 (57). As to the pro-tumor ability, both IL-1β and IL-1α are found to contribute to tumor angiogenesis and invasiveness in the process of PCa progression (58). Additionally, IL-1α and IL-1β are found to be able to reprogram AR+ PCa cells to AR- PCa cells, resulting in CRPC and treatment resistance (59). It was reported that IL-1α can interact with IL-6 to generate PSMA/PSA prostate clones (60). Two ETS family members associated with PCa malignancy and poor prognosis for patients, epithelium-specific ETS (E26 transformation-specific) and ESE1 (or E74-like factor (ELF3), can be activated by IL-1β through NF-κB pathway (61). IL-1β can also induce the expression of endothelin 1 (ET-1) and matrilysin 1, which are implicated in PCa progression (62, 63). IL-1β was also reported to induce IL-8 through the MAPK pathway to promote PCa proliferation (64). IL-1RA is a specific receptor antagonist, and its expression is negatively related to Gleason score. Studies demonstrated that IL-1RA can inhibit the activity of IL-1α and IL-1β (65). Many studies have reported that IL-1RA can reduce tumor-mediated inflammation and invasion (65, 66).

IL-18, which is a proinflammatory cytokine structurally similar to IL-1β, is significantly associated with poor prognosis in PCa (67). IL-18 also exerts a dual function of promoting or suppressing tumor progression. Similar to IL-1β, IL-18 could also promote tumor progression by regulating the myeloid differentiation factor 88 (MyD88)/NF-κB signaling pathway (68). Various evidence firstly demonstrated that IL-18 is closely associated with tumor growth. This conclusion came from that elevated expression of IL-18 by tumor cells were observed in the serum of PCa patients (69). A high level of IL-18 was found to be associated with angiogenesis, tumor cell migration, and metastasis (67). It was reported that IL-18 can also mediate evasion of anti-tumor immune response (55). While IL-18 also has a tumor-suppressive effect that it was shown to activate CD4+T cells and NK cells and suppress the progression of tumor metastasis. Multiple shreds of evidence show that IL-18 can activate macrophages to release IFN-γ and neutrophils to produce TNF. However, elevated expression of IL-18 binding protein (IL-18BP), an inhibitory IL-18 receptor, was found to be associated with resistance to anti-tumor immune responses and correlates with poor prognosis in patients with PCa (70).

Previous studies widely acknowledged that IL-33 functions as immune surveillance in the immune system, which was found to regulate homeostasis, and works as an alarmin in response to infection or stress (71). And the loss of IL-33 expression was found to be associated with recurrence and metastatic immune evasion (52). Apart from its anti-tumor effect, accumulative studies have demonstrated its crucial role in malignancy (72). It is involved in many processes of tumor progression, such as oncogenesis, tumor growth, angiogenesis, metastasis and immune evasion (72). The IL-33/ST2 axis is emerging as a potent modulator of the TME. It was found to remodel the TME to promote malignancy or induce tumor regression by recruiting a cohort of immune cells (73).

Research on IL-36 mainly focuses on IL-36γ. IL-36γ was shown to strengthen the effector functions of CD8+T cells, NK cells, and γδ T cells, making the tumor microenvironment favor tumor destruction, and ultimately to have profound anti-tumor effects, suppressing both tumor growth and metastasis (74). It was also reported that IL-36γ has the potential to decrease MDSCs and increase IFN-positive CD4+ and CD8+ T cells (75).

IL-37 exerts a negative effect on cancer cell proliferation and invasion through STAT3 signaling (76, 77). IL-37 was reported to suppress the activation of NF-κB and MAPK, and negatively regulate proinflammatory cytokines and pro-tumor signaling pathways (78). However, high levels of IL-37 in the serum of patients with certain cancers such as ovarian cancer were found to be associated with poor prognosis, poor overall survival and progression-free survival (79), while the connection of IL-37 with PCa is unclear yet. IL-37 was also found to downregulate the expression of TIM3 on canonical NK cells, which plays a vital role in the anti-tumor immune response by inhibiting the immune suppression mediated by Treg. Blocking IL-37 was proved to eliminate Treg suppression of canonical NK cells (80). Similar to the molecular structures of IL-1RA and IL36Ra, IL-38 is considered as the antagonist of the IL-1 receptor. IL-38 mainly exerts its function by inhibiting Th17 (75).

## IL-1 family and bone metastasis of PCa

**Figure 3:**
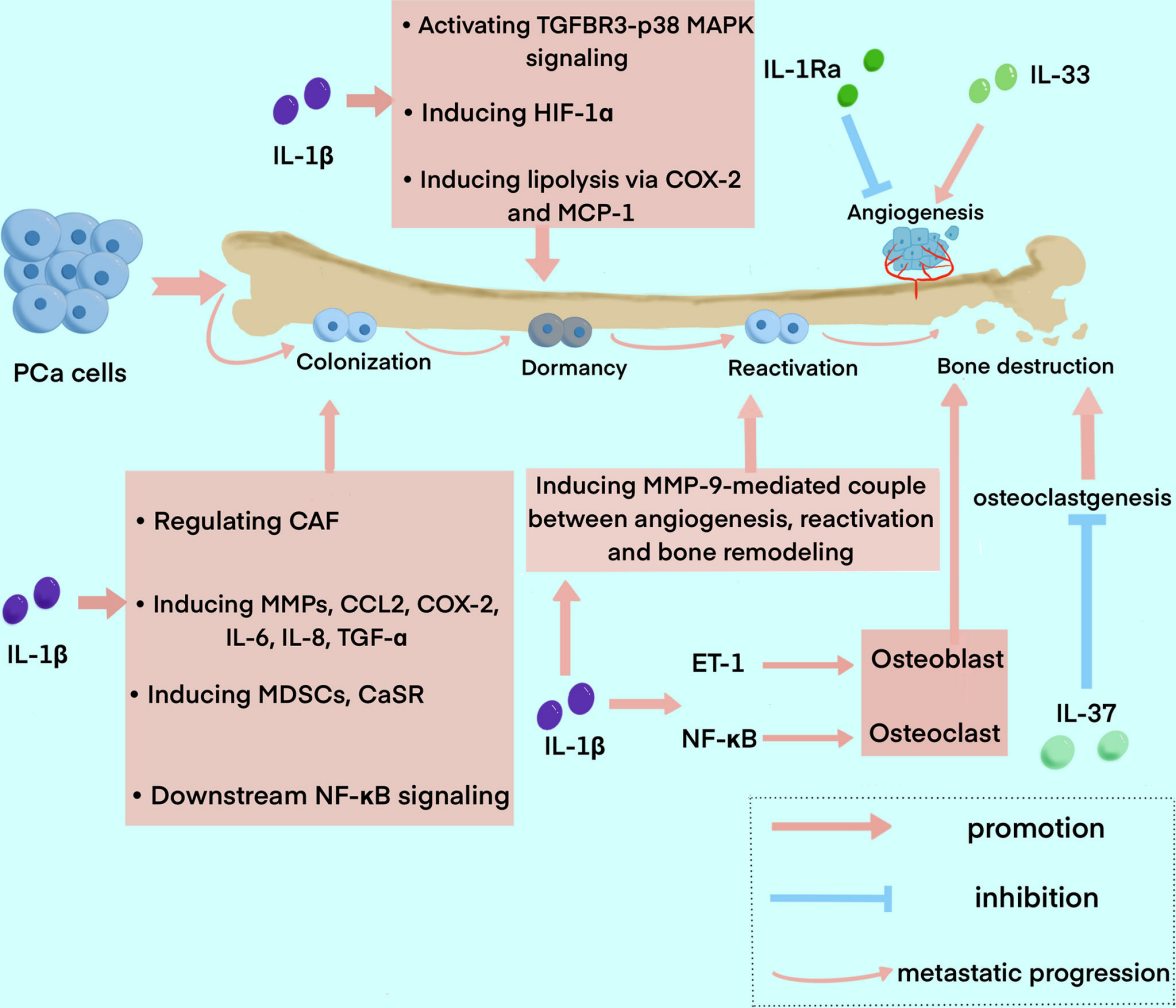
The roles of IL-1 family in the process of bone metastasis in PCa. The diagram summarized the major functions of IL-1 family exert in the process of bone metastasis, including colonization, dormancy, reactivation, and bone destruction. IL1β is widely involved in the whole process, while IL-1Ra, IL-33, and IL-37 mainly play a role in angiogenesis and bone destruction.

Bone metastasis of PCa goes through a series of processes, including EMT, formation of a pre-metastatic niche, bone colonization, metastatic dormancy, reactivation, and reconstruction in the bone niche (21). Many pieces of research have demonstrated that IL-1 families are closely involved in this process (**Figure 3**).

Recent research on the role IL-1 family plays in PCa bone metastasis mainly focus on IL-1β. IL-1β was found to be able to transform the bone stroma into a niche favorable for metastasis, and promote bone colonization of metastatic tumor cells (81). It was reported that IL-1β secreted by metastatic PCa cells promotes colonization and progression to the bone with neuroendocrine features (82). Previous studies elucidated that the formation of CAF and bone stromal alterations, as the key drivers of early colonization in IL-1β-mediated bone metastasis, can be regulated by many factors. For instance, COX-2, S100A4 and TNF13 expressed in human bone mesenchymal could be upregulated by IL-1β secreted by metastatic PCa cells (81). And the elevation of CAF can be blocked by anakinra, an IL-1R antagonist (83). IL-1β was also found to regulate the expression of tumor-related genes in the bone stroma (81). Studies also reported that IL-1β can induce the expression of many other factors related to bone metastasis and inflammation, such as matrix MMPs, CCL2, COX-2, IL-6, IL-8 and TGF-α (84, 85). MMPs induced by IL-1β can also promote the development of a metastasis niche to other factors such as integrin αvβ3, an adhesion molecule (86). IL-1β can also induce MDSCs, which is one of the major suppressors of antitumor immunity and a crucial element in cancer progression and metastasis (87). A study also showed that IL-1β stimulates CaSR expression, which may impact calcium homeostasis, thus influencing the progression and bone metastasis of PCa (88). It has been shown that a direct relationship between bone marrow-derived IL-1β and NF-κB, one of its main downstream transcription factors, favors PCa stem cell colonization and outgrowth within the bone (89, 90). Furthermore, the significant interaction of NF-κB/STAT3 with downstream inflammatory pathways assures a constant positive feedback, resulting in signal amplification of PCa cells (91).

After cancer cell colonization, TGF-β2 induced by IL-1β promotes PCa cell dormancy *via* activation of TGFBR3–p38 MAPK signaling (92). Additionally, IL-1β can upregulate hypoxia-inducible transcription factor-1α(HIF-1α), which transcripts VEGF and COX-2 and promotes angiogenic activity, thus providing a microenvironment favorable for tumor growth in bone metastatic sites (93, 94). Furthermore, it was demonstrated that metastatic PCa cells interact with bone marrow adipocytes to promote IL-1β expression, and in turn, IL-1β upregulates COX-2 and MCP-1 to induce lipolysis and an inflammatory phenotype. This could explain the mechanism of the insensitivity of PCa to Docetaxel (84). In addition, paracrine factors from bone marrow stromal cells (BMSC) can also cause apoptosis of metastatic PCa cells in the bone marrow, while IL-1β-induced p62/SQSTM1 is a fundamental factor for cell survival in this process as an apoptosis protector (95, 96).

Beyond the process of bone colonization and metastatic dormancy, metastatic tumor cells are reactivated and result in bone reconstruction. The role the IL-1 family plays in the reactivation of metastatic dormant cells in bone is unclear, yet, while the MMP-9-mediated couple between angiogenesis and bone resorption may have an implication for reactivation, and the process is closely associated with IL-1β (97). Furthermore, it was found that increased osteoclast activity is also related to the reactivation of dormant cells (97). Bone metastasis of PCa is usually osteoblastic. On the contrary, previous research have shown that IL-1, RANKL and TNF-α are implicated in the osteolytic phenotype of PCa reported in some cases (98, 99). ET-1 induced by IL-1β is associated with the osteosclerotic lesion, which suppresses DKK-1, the inhibitor of Wnt (48, 100). And NF-κB activated by IL-1β promotes osteolytic lesions by inducing osteoclast cells through pathways such as GM-CSF and RANKL (101). In one study, IL-1β was reported to have the potential to inhibit metastasis by decreasing the chemotaxis of PCa bone metastasis and can inhibit PCa cell growth and bone metastasis with other cytokines such as TNF-α (102).

Microenvironmental IL-1β has also been shown in studies to enhance breast cancer metastatic colonization in the bone by activating Wnt signaling (103, 104). However, the role of IL-1 in the Wnt signaling pathway in PCa is unclear, yet, which could be a possible target to inhibit IL-1 signal transduction.

IL-1RA mainly exerts anti-tumor function, which was reported to inhibit bone metastasis by reducing tumor inflammation, angiogenesis, and immune suppression (65, 66). IL-1RA was found to be associated with a clinical pathological feature, so that IL-1RA is inversely correlated with Gleason score and pathological stage (105). The tumor burden was significantly reduced in animals treated with IL-1RA (81). A study demonstrated that inhibition of IL-6 expression by IL-1RA increases progression-free survival (75).

There is no concrete evidence of the role IL-18 plays in bone metastasis up to now, while considering its association with angiogenesis, tumor cell migration, and metastasis, the following studies should focus on the function this cytokine has in bone metastasis (55).

IL-33 was found to play a role in immune surveillance, and its absence can lead to immune evasion, which is a key process in bone metastasis (52). IL-33 was also reported as a key driver of treatment resistance in PCa, which could be a possible target for upregulating sensitivity to cancer treatment (106). Research reported that reduced IL-33 expression in PCa is connected with metastasis (52). As accumulative evidence proved that IL-33 plays a role in malignancy, such as angiogenesis, metastasis, and immune evasion, its dual role in bone metastasis is necessary to be discovered (72).

IL-37 shows a negative effect on cancer cell proliferation and invasion through the STAT3 signaling pathway (76). A study demonstrated that IL-37 inhibits osteoclastogenesis and bone resorption mediated by RANKL or LPS and relieves inflammatory bone destruction and bone resorption (107). In the study, IL-37 was reported to affect the activation of NF-κB and IκBα in response to RANKL, thus inhibiting osteoclast formation. Furthermore, IL-37 can also decrease the phosphorylation of inhibitors of IκBα and NF-κB(p65) and the expression of nuclear factor of activated T cells c1 (107).

## Possible treatment therapies targeted at IL-1 family

The majority of patients with bone metastasis cannot be diagnosed until they suffer from SREs, which is usually the late stage of metastasis, thus missing the optimal time for treatment. Therefore, valid early biomarkers are urgently needed. IL-1α is reported to show a negative correlation with biochemical advancement, IL1β is linked to clinical T stage, and IL-1RA has an inverse correlation with Gleason score (83). IL-18 is also associated with poor prognosis and decreased survival (67). Furthermore, as the reduced expression of IL-33 in PCa is connected with metastasis, it could be a possible biomarker to detect the metastasis and prognosis of PCa patients. Thus, the IL-1 family can be possible biomarkers for early diagnosis to carry out the timely treatment.

Even though a number of current bone therapeutic agents can successfully prevent PCa from spreading to the bones, only a small percentage of PCa patients have long-lasting benefits from them. Consequently, there is an urgent need for the development of innovative therapeutic approaches to inhibit the bone metastasis of PCa. According to the findings that IL-1 plays a vital role in tumorigenesis, including proliferation, angiogenesis, and metastasis, it could be an effective target of chemotherapy. **Table 1** summarized the major functions and the mechanism of the IL-1 family.

**Table 1:** Role of IL-1 family in bone metastasis and possible therapy which is currently known.

Cytokines	Function	Mechanism	Treatment
IL-1α	Unknown		
IL-1β	Pro-tumour	Establishing a pre-metastatic niche (81)	Knockdown: Significantly inhibited bone metastasis (82) Make PCa cells sensitive to chemotherapy (84)
		Dormancy (82)	
		Reactivation (93)	
		Angiogenesis (93)	
		Bone remodeling (97)	
	Anti-tumour	Decrease the chemotaxis of PCa (102)	N/A
IL-1RA	Anti-tumour	Reduce tumour inflammation (65)	Anakinra (approved) (108) Rilonacept (under experiment) (109)
		Angiogenesis (65)	
		Immune recruitment (66)	
IL-18	Unknown		
IL-33	Pro-tumour	Treatment resistance (106)	N/A
		Angiogenesis (72)	
		Metastasis (72)	
		Immune evasion (72)	
	Anti-tumour	Immune surveillance (52)	N/A
IL-36	Unknown		
IL-37	Anti-tumour	Inhibition of osteoclastogenesis and bone resorption (107)	N/A
IL-38	Unknown		

N/A - not applicable

Anakinra, the approved IL-1RA medicine, was proved to reduce tumor growth and metastasis in preclinical PCa models. Treatment with anakinra creates an immunological-friendly milieu, making CRPC more susceptible to immune checkpoint blockade (108). At the same time, IL-1RA upregulation induced by a combination of immune checkpoint inhibition and MDSC-targeted treatment is essential for reducing MDSC infiltration, which is another key factor in metastasis. Additionally, the IL1β-neutralizing human antibody, Canakinumab, the human antibody targeting IL-1α, MABp1, and Rilonacept, a dimeric fusion protein made up of the ligand-binding domain of human IL-1R and IL-1RAcP are all under preclinical experiment and show effectiveness to different degrees (109). However, their effects on inhibiting bone metastasis are unclear now.

As IL-1β is widely involved in the process of bone metastasis, including the establishment of a pre-metastatic niche, bone colonization, metastatic dormancy, reactivation, and bone reconstruction, it could be an ideal target to prevent bone metastasis. Research reported that the knockdown of IL-1β significantly inhibited the bone progression of highly metastatic PCa cells (82). Inhibiting IL-1β was reported to promote cell apoptosis induced by BMSC paracrine factors (82). As Increased reactive oxygen species (ROS) along with inflammation are involved in PCa, the antioxidant arbutin was found to decrease the expression of IL-1β (110). Given that IL-1β mediates the insensitivity of PCa to docetaxel, inhibiting IL-1β could make PCa cells sensitive to chemotherapy again (84). However, IL-1α and IL1β show inverse effects in each stage of the malignant process, so which and when to target should be precisely answered (109). Similarly, IL-18 and IL-33 also exert dual functions in the process of bone metastasis in different stages, thus having a deep insight into the specific function these cytokines play in different phases is fundamental to subsequent treatment. Given that IL-37 shows the feature of inhibiting osteoclastogenesis, activation of IL-37 could be another possible therapy. In addition, combining IL-1 blockade with immune checkpoint inhibitors, such as anti-PD-1 and anti-CTLA4, has shown effectiveness in breast cancer, and their combination in PCa is also worth researching as the IL-1 family exert a vital role in immune response and immune microenvironment (111).

## Conclusions

As we reviewed above, PCa progression and metastasis have a close relationship with inflammatory cytokines. It is well known that the IL-1 family plays a crucial role in regulating the progression and bone metastasis of PCa. IL-1 family always exerts the unique functions in the bone metastasis of PCa. IL-1β could promote bone colonization of metastatic tumor cells through transforming the bone stroma into a niche. Furthermore, IL-1β could promote osteolytic activity and induce the dormancy, angiogenesis, and reactivation of tumor cells in PCa. IL-1RA could probably suppress bone metastasis through regulating inflammation, angiogenesis and immune response in PCa. To date, the role of IL-18 in bone metastasis of PCa is still debated. Recent studies have revealed that IL-33 could play a dual role in bone metastasis of PCa through regulating the immune surveillance and the progression of tumor. On the contrary, IL-37 could significantly inhibit the proliferation and invasion of tumor cells in PCa. Meanwhile, IL-37 could reduce bone resorption through inhibiting osteoclastogenesis. Considering the pivotal roles of IL-1 family in bone metastasis of PCa, the development of the novel reliable molecular targeted agents to provide individualized clinical treatments for PCa is urgently needed. For instance, Canakinumab, IL-1β-neutralizing human antibody, and MABp1, the IL-1α inhibitor, have demonstrated clinical benefits for PCa patients with bone metastasis. The inhibition of IL-1β has proven considerable efficacy in suppressing tumor progression through inhibiting angiogenesis and osteolytic activity of PCa cells. In addition, Anakinra, as the approved IL-1RA medicine, is widely applied to reduce tumor growth and bone metastasis in PCa.

In conclusion, despite prior research studies have validated the efficacy of IL-1 family-directed agents in bone metastasis of PCa, the exact mechanisms of IL-1 family, such as IL-1α, IL-18, IL-33, IL-38, are urgently needed to be fully elucidated. It is still unknown whether the combination of immune checkpoint inhibitor therapy and IL-1 family-directed agents would probably demonstrates superiority over immune checkpoint inhibitors monotherapy in bone metastasis of PCa.

## Author contributions

YT and MM contributed to the design and conception of the review, YC, TJ, ZH, and QH wrote sections of the manuscript. All authors contributed to manuscript revision, read, and approved the submitted version.

## Funding

This study was supported by grants from the National Natural Science Foundation of China (NSFC) (82103532).

## Conflict of interest

The authors declare that the research was conducted in the absence of any commercial or financial relationships that could be construed as a potential conflict of interest.

## Publisher’s note

All claims expressed in this article are solely those of the authors and do not necessarily represent those of their affiliated organizations, or those of the publisher, the editors and the reviewers. Any product that may be evaluated in this article, or claim that may be made by its manufacturer, is not guaranteed or endorsed by the publisher.
